# The association between physical activity and adolescent idiopathic scoliosis: improper sitting posture as a mediator

**DOI:** 10.3389/fped.2026.1884485

**Published:** 2026-07-31

**Authors:** Lei Wang, Panpan Ge, Hongyu Wang

**Affiliations:** 1 Department of Physical Education, Anhui Wenda University of Information Engineering, Hefei, China; 2 College of Humanities and Health Sciences, Bengbu Medical University, Bengbu, China

**Keywords:** adolescent, idiopathic scoliosis, improper sitting posture, mediation analysis, physical activity

## Abstract

**Background:**

The association between physical activity (PA) and adolescent idiopathic scoliosis (AIS) remains unclear. This study investigates whether improper sitting posture mediates the relationship between PA and AIS in cross-sectional analyses.

**Methods:**

This study utilized data on adolescents collected across 9 provinces in China in 2021, involving 3,536 participants for cross-sectional analysis. A multivariate logistic regression model was used to evaluate the associations among PA, improper sitting posture, and AIS. Mediation analyses assessed the mediating role of improper sitting posture in the association between PA and AIS. Subgroup analyses were performed to assess heterogeneity across groups.

**Results:**

In the cross-sectional analysis, PA (OR = 0.985, 95%CI: 0.981–0.990, *P* < 0.001) was negatively associated with AIS, and improper sitting posture (OR = 1.989, 95%CI: 1.664–2.376, *P* < 0.001) was positively associated with AIS. Improper sitting posture partially mediated this relationship, with a mediation effect of −0.0011 (95% CI: −0.0018 to −0.0005), representing 7.10% of the overall effect variability.

**Conclusion:**

PA was negatively associated with improper sitting posture and AIS, and improper sitting posture was positively associated with AIS. Improper sitting posture partially mediated the association between PA and AIS. These results suggest that PA and the avoidance of improper sitting posture may help prevent AIS.

## Introduction

Adolescent idiopathic scoliosis (AIS) is a three-dimensional structural deformity of the spine that arises during the growth spurt, typically between 10 and 18 years of age, and is defined radiographically by a Cobb angle ≥ 10°; it represents the most common spinal deformity in adolescents despite an incompletely understood etiology (1). AIS, as the most common form of scoliosis, accounts for 84%–89% of all types of scoliosis (2). Globally, AIS is recognized as an important public health concern, with a reported prevalence of approximately 0.47%–5.20%, although substantial geographic variation exists (3). An adolescent scoliosis screening program carried out in Shenzhen, China, between 2018 and 2019 revealed an estimated prevalence of 3.2% (4). AIS can result in a range of adverse consequences such as back pain (5), poor self-image (6), increased psychological stress (7), and even cardiopulmonary damage in severe cases (8), which significantly impacting the quality of life. Therefore, elucidating the determinants and modifiable pathways of AIS is essential for informing early identification and targeted prevention strategies.

Physical activity (PA) is defined as any bodily movement produced by skeletal muscles that results in energy expenditure (9). Previous studies have suggested that lower levels of PA are associated with a higher prevalence of spinal deformities, including AIS (10). Insufficient PA is associated with reduced trunk muscle strength and impaired neuromuscular control, which can weaken spinal stability and increase susceptibility to postural asymmetry (11). Regular PA can enhance muscle strength, neuromuscular coordination, and spinal alignment in adolescents, thereby contributing to the maintenance of normal spinal biomechanics and potentially mitigating the progression of AIS (12). Elucidating the role of PA in AIS may help identify modifiable behavioral factors relevant to adolescent spinal health.

Improper sitting posture is generally defined as sustained non-neutral spinal alignment, manifested as deviations from recommended reading-writing distances, inappropriate pen-holding angle, and reading under non-upright conditions, which may increase mechanical stress on the musculoskeletal system (13). PA, as an overall volume reflecting habitual movement exposure, may play a protective role against such postural behavior (14). Higher levels of PA have been associated with improved musculoskeletal fitness, including trunk muscle endurance and postural control, which are essential for maintaining proper sitting alignment (15). In addition, greater PA is linked to reduced sedentary time and screen-based behaviors, thereby decreasing prolonged exposure to static, maladaptive sitting positions (16). Through these neuromuscular and behavioral pathways, adequate PA may help prevent the development of improper sitting posture in adolescents. Clarifying this relationship is therefore important for understanding modifiable behavioral factors underlying postural health.

Improper sitting posture is strongly associated with a significantly increased risk of AIS abnormalities and has been increasingly recognized as a risk factor for AIS (17). Sustained non-neutral spinal alignment, such as slumped or asymmetric sitting, can lead to uneven mechanical loading across the vertebral bodies, which may influence spinal growth and deformity development in accordance with growth modulation principles (18). In addition, prolonged postural asymmetry is associated with muscular imbalance and altered trunk biomechanics, further compromising spinal stability and promoting curve initiation or progression (19). Adolescents may be particularly vulnerable to these effects due to ongoing musculoskeletal development and the high proportion of time spent in seated activities during school hours. Therefore, given the established links between PA, postural alterations, and spinal stress, improper sitting posture may mediate the relationship between PA and AIS. However, existing studies have largely examined PA and sitting posture as independent factors, and little is known about their interrelationship in the context of AIS, particularly whether improper sitting posture serves as a behavioral pathway linking PA to AIS. Improper sitting posture may be a behaviorally modifiable and biomechanically proximate pathway between PA and AIS. Greater PA volume is associated with less sedentary exposure and better trunk muscle endurance and proprioceptive control, which may facilitate neutral spinal alignment during prolonged sitting (20, 21). Insufficient PA may therefore relate to habitual maladaptive sitting, which in turn might contribute to asymmetric loading on the growing spine and suggest a potential mechanistic link to AIS. Given the above background, we hypothesized that improper sitting posture mediates the association between PA and AIS.

This study examined the associations between PA and improper sitting posture with AIS across nine provinces in China, with baseline data collected in 2021. We tested three *a priori* hypotheses: (H1) PA is negatively associated with AIS; (H2) improper sitting posture is positively associated with AIS; and (H3) improper sitting posture mediates the association between PA and AIS. The findings elucidate behavioral pathways underlying AIS and support the development of targeted, school-based interventions.

## Methods

### Study design and population

**Figure 1:**
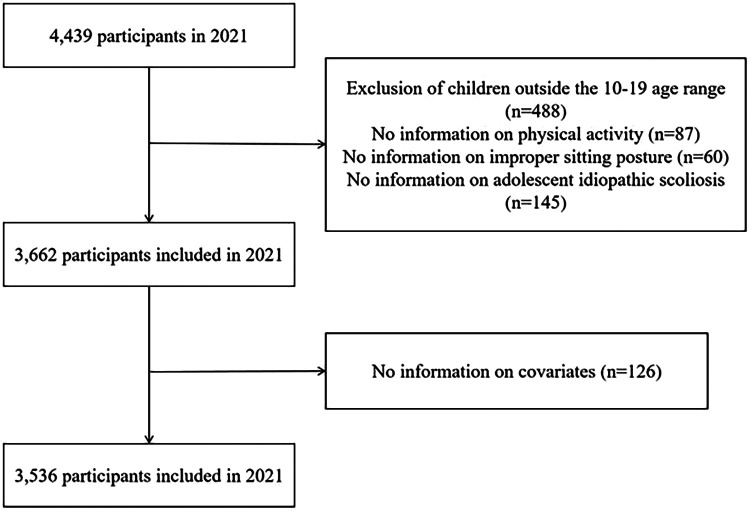
Flowchart of the participants selection process.

The study was conducted in nine provinces in China in 2021. A total of 4,439 adolescents were surveyed. Figure 1 shows the selection process of the study population. At baseline, 4,439 participants were assessed for the baseline analysis. Participants were excluded based on the following criteria to ensure methodological accuracy: children outside the 10–19 age range (*n* = 488), incomplete PA data (*n* = 87), missing data on improper sitting posture (*n* = 60), missing AIS data (*n* = 145), and incomplete covariate information (*n* = 126). A total of 3,536 individuals were retained for the cross-sectional analysis.

### Assessments

#### Physical activity

PA was assessed using the Physical Activity Rating Scale-3 (PARS-3), a validated self-report instrument originally developed by Hashimoto and subsequently culturally adapted and psychometrically refined for the Chinese population by Liang Deqing et al. (22). The PARS-3 evaluates three core dimensions of PA over the preceding month—intensity, duration, and frequency—each rated on a 5-point ordinal scale.

Intensity reflects the physiological demand of the activity: 1 = mild (e.g., leisurely walking or radio calisthenics); 2 = light (e.g., recreational table tennis or slow jogging); 3 = moderate and sustained (e.g., cycling at a steady pace or continuous jogging); 4 = vigorous but intermittent (e.g., basketball or singles tennis); 5 = vigorous and sustained (e.g., competitive running or lap swimming).

Duration denotes the average length of a single session, in minutes: 1 = <10 min; 2 = 11–20 min; 3 = 21–30 min; 4 = 31–59 min; 5 = ≥60 min.

Frequency indicates the typical number of PA sessions per month: 1 = ≤1 session/month; 2 = 2–3 sessions/month; 3 = 1–2 sessions/week (i.e., 4–8 sessions/month); 4 = 3–5 sessions/week (i.e., 12–20 sessions/month); 5 = approximately daily (i.e., ≥21 sessions/month).

The total PA score is calculated as the product of the three dimension scores (intensity × duration × frequency), yielding a continuous composite score ranging from 5 to 75. Consistent with established PARS-3 scoring guidelines, participants are classified into three PA volume categories: low (score ≤ 19), moderate (score 20–42), and high (score ≥43).

#### Adolescent idiopathic scoliosis

A diagnosis of AIS was determined through a two-phase screening protocol (1). Phase I comprised a physical examination of the spine, including inspection for shoulder asymmetry and assessment of spinal curvature. This phase also incorporated the Adams forward bend test, during which the participant was asked to stand with feet together, bend forward slowly with the trunk flexed approximately 90 degrees, and allow the arms to hang downward with palms opposed. A scoliometer was used to measure the angle of trunk rotation at the most prominent site of rib or lumbar prominence. An angle of trunk rotation of 5 degrees or greater was considered screen-positive and indicated the need for further radiographic evaluation. Phase II involved full-length standing anteroposterior radiography of the whole spine. The Cobb angle was measured on the radiograph according to the standard method by identifying the upper end vertebra and lower end vertebra of the curvature, drawing lines along the superior endplate of the upper end vertebra and the inferior endplate of the lower end vertebra, and then measuring the angle between the perpendiculars to these two lines. A Cobb angle of 10 degrees or greater was required to confirm a definitive diagnosis of AIS. This two-phase protocol was consistent with the national guidelines for scoliosis screening in China as well as the international definition recommended by the Scoliosis Research Society (1).

#### Improper sitting posture

This data was collected in 2021. The improper sitting posture among adolescents was defined using three evidence-informed criteria from China's Ministry of Education for proper reading and writing posture: (1) upright seated position, with eye-to-book distance of 33–35 cm, chest-to-desk distance of ∼1 fist, and finger-to-pen-tip distance of ∼3 cm; (2) appropriate pen-holding angle—40°–50° for pencils or pens, and near-vertical for brush pens; and (3) avoidance of reading while head-tilted, supine, walking, or in moving vehicles or vessels. Adolescents who violated one or more of the above criteria were considered as having improper sitting posture.

### Validity and reliability of the questionnaire

Reliability and validity of all measurement instruments were rigorously evaluated. As summarized in Table 1, internal consistency was assessed using Cronbach's *α*: PA (*α* = 0.645). Coefficients met or exceeded the conventional threshold of 0.6, supporting acceptable reliability.

**Table 1:** Reliability analysis.

Variables	Number of items	Cronbach's *α* value
Physical activity	3	0.645

Construct validity was examined via exploratory factor analysis prerequisites: the Kaiser–Meyer–Olkin (KMO) measure of sampling adequacy and Bartlett's test of sphericity (Table 2). KMO values were 0.614 for PA, indicating sufficient variable correlations for factor analysis. Furthermore, Bartlett's test yielded statistically significant results (*p* < 0.001), confirming that the correlation matrices were sufficiently intercorrelated to justify scale-level interpretation.

**Table 2:** KMO and Bartlett's sphericity test.

Variables	KMO and Bartlett's sphericity		
Physical activity	KMO value		0.614
	Bartlett's sphericity test	Approximate chi-square	1,556.403
		*df*	3
		*p*	<0.001

KMO, Kaiser-Meyer-Olkin.

### Confirmatory factor analysis

Confirmatory factor analysis was performed using AMOS 24.0, and the model demonstrated acceptable fit indices: the ratio of chi-square to degrees of freedom (*χ*^2^/df) was 3.988, the root mean square error of approximation (RMSEA) was 0.074, the goodness-of-fit index (GFI) was 0.901, the comparative fit index (CFI) was 0.853, the incremental fit index (IFI) was 0.847, and the normed fit index (NFI) was 0.874. These values indicate that the hypothesized model adequately represents the underlying structure of the observed variables and their relationships with the latent constructs.

### Control variables

The baseline questionnaire collected demographic and health data, including age and sex (male or female). Age was recorded and grouped for analysis. Anthropometric measurements, specifically height and weight, were collected using standardized protocols to calculate body mass index (BMI). Participants were classified into four BMI categories according to World Health Organization (WHO) criteria: underweight (BMI < 18.5 kg/m^2^), normal weight (BMI: 18.5–24.9 kg/m^2^), overweight (BMI: 25.0–29.9 kg/m^2^), and obese (BMI ≥ 30.0 kg/m^2^). Daily sleep duration was also assessed and categorized into three groups: ＜7 h, 7–9 h, and ＞9 h.

### Statistical analysis

Statistical analyses were conducted in R (v4.3.3). Descriptive statistics summarized participant characteristics: continuous variables as mean ± SD; categorical variables as frequency (percentage). Spearman rank correlations assessed bivariate associations among key variables. Multivariable logistic regression models evaluated the associations of PA and improper sitting posture with AIS, yielding odds ratios (ORs). Two models were fitted: an adjusted model (adjusted for sex, age, BMI, and daily sleep duration) and an unadjusted model. Subgroup analyses explored effect heterogeneity across demographic, behavioral, and health-related strata. Owing to the number of statistical tests we performed, a Bonferroni correction was used for multiple testing. Mediation analysis—using the causal steps approach—tested whether improper sitting posture mediated the association between PA and AIS. Adjusted models assessed: (1) the association between PA and AIS; (2) the association between PA and improper sitting posture; and (3) the association between improper sitting posture and AIS. Mediation effects were estimated using bootstrapped 95% CIs (1,000 replications); statistical significance was defined as a 95% CI excluding the null value (OR = 1).

## Result

### Characteristics of the study participants at baseline

A total of 3536 participants were included (Table 3), of whom 163 (4.6%) were identified with AIS. Compared with the non-AIS group (*n* = 3,373), the AIS group (*n* = 163) showed significant differences. In terms of demographic characteristics, sex distribution differed significantly (*P* = 0.020), with a higher proportion of females in the AIS group (5.4% vs. 3.8% in males). Age distribution also showed significant differences (*P* = 0.038), with the highest proportion of AIS observed aged 13–15 years (5.4%), compared with those aged 10–12 years (3.5%) and 16–18 years (3.7%). Regarding anthropometric measures, BMI categories differed significantly between groups (*P* < 0.001). The AIS group had a higher proportion of overweight individuals (8.5% vs. 4.4% in normal weight) and a lower proportion of underweight individuals (1.7%), while the prevalence among obese adolescents was 3.8%. All PA indicators showed significant differences (all *P* < 0.001). Compared with the non-AIS group, the AIS group had lower levels of PA, including reduced intensity (1.28 ± 0.05 vs. 1.29 ± 0.02), shorter duration (1.20 ± 0.05 vs. 1.23 ± 0.02), lower frequency (1.14 ± 0.04 vs. 1.18 ± 0.02), and lower overall PA (19.25 ± 0.74 vs. 23.64 ± 0.44). In addition, the improper sitting posture was significantly higher in the AIS group compared with the non-AIS group (2.09 ± 0.58 vs. 1.93 ± 0.46, *P* < 0.001).

**Table 3:** Baseline characteristics of selected participants (*N* = 3,536).

Variables	Total	Non-AIS	AIS	*P-value*
Total	3,536	3,373	163	
Sex, *n* (%)				0.020
Male	1,790 (50.6)	1,722 (96.2)	68 (3.8)	
Female	1,746 (49.4)	1,651 (94.6)	95 (5.4)	
Age, *n* (%)				0.038
10～12 years old	1,074 (30.4)	1,036 (96.5)	38 (3.5)	
13～15 years old	2,000 (56.6)	1,892 (94.6)	108 (5.4)	
16～19 years old	462 (13.1)	445 (96.3)	17 (3.7)	
BMI, *n* (%)				＜0.001
Underweight	770 (21.8)	757 (98.3)	13 (1.7)	
Normal weight	1,630 (46.1)	1,558 (95.6)	72 (4.4)	
Overweight	740 (20.9)	677 (91.5)	63 (8.5)	
Obese	396 (11.2)	381 (96.2)	15 (3.8)	
Daily sleep duration, *n* (%)				0.304
＜7 h	1,050 (29.7)	996 (94.9)	54 (5.1)	
7–9 h	2,242 (63.4)	2,140 (95.5)	102 (4.5)	
＞9 h	244 (6.9)	237 (97.1)	7 (2.9)	
Intensity of physical activity	2.59 ± 1.29	2.62 ± 1.29	2.12 ± 1.21	<0.001
Duration of physical activity	1.89 ± 1.23	1.90 ± 1.23	1.56 ± 1.17	<0.001
Frequency of physical activity	3.31 ± 1.18	3.32 ± 1.18	2.96 ± 1.10	<0.001
Physical activity	21.10 ± 13.01	21.51 ± 13.18	12.66 ± 12.21	<0.001
Improper sitting posture	1.96 ± 0.49	1.96 ± 0.50	2.01 ± 0.44	<0.001

BMI, body mass index; AIS, adolescent idiopathic scoliosis.

### The association between PA and improper sitting posture with AIS

Table 4 presents the results of the regression analysis examining the associations of PA and improper sitting posture with AIS. In the unadjusted model, higher PA was inversely associated with AIS (OR = 0.986, 95% CI: 0.981–0.990, *P* < 0.001), with consistent inverse associations observed across all three PA dimensions: intensity (OR = 0.817, 95% CI: 0.764–0.873), duration (OR = 0.864, 95% CI: 0.806–0.927), and frequency (OR = 0.823, 95% CI: 0.766–0.884) (all *P* < 0.001). In contrast, improper sitting posture was positively associated with AIS (OR = 1.960, 95% CI: 1.644–2.337; *P* < 0.001). After adjustment for sex, age, BMI, and daily sleep duration, these associations remained statistically significant. PA retained its inverse association with AIS (OR = 0.985, 95% CI: 0.981–0.990, *P* < 0.001), and all dimensional measures continued to show robust inverse effects—intensity (OR = 0.818, 95% CI: 0.761–0.879), duration (OR = 0.865, 95% CI: 0.804–0.932), and frequency (OR = 0.814, 95% CI: 0.757–0.876) (all *P* < 0.001); improper sitting posture also remained significantly positively associated (OR = 1.989, 95% CI: 1.664–2.376; *P* < 0.001). Collectively, these findings indicate that lower levels of PA and greater prevalence of improper sitting posture are independently associated with increased odds of AIS.

**Table 4:** Regression analysis of physical activity and improper sitting posture on adolescent idiopathic scoliosis.

Variables	No.	Unadjusted		Adjusted	
		*OR (95% CI)*	*p*	*OR(95% CI)*	*p*
Physical activity	3,536	0.986 (0.981, 0.990)	<0.001	0.985 (0.981, 0.990)	<0.001
Intensity of physical activity	3,536	0.817 (0.764, 0.873)	<0.001	0.818 (0.761, 0.879)	<0.001
Duration of physical activity	3,536	0.864 (0.806, 0.927)	<0.001	0.865 (0.804, 0.932)	<0.001
Frequency of physical activity	3,536	0.823 (0.766, 0.884)	<0.001	0.814 (0.757, 0.876)	<0.001
Improper sitting posture	3,536	1.960 (1.644, 2.337)	<0.001	1.989 (1.664, 2.376)	<0.001

After adjusting for Sex, age, BMI and daily sleep duration.

Based on the subgroup analyses of the relationship between PA and AIS (Supplementary Table 1) and between PA and improper sitting posture (Supplementary Table 2), no significant interaction effects were observed across all variables (all *P* for interaction > 0.0125). Supplementary Table 3 shows that BMI significantly modified the association between improper sitting posture and AIS (interaction for *P* = 0.008).

### Improper sitting posture mediated the association between PA and AIS

Table 5 presents the bivariate associations among PA, improper sitting posture, and AIS. PA volume was inversely associated with both improper sitting posture (*r* = −0.100, *P* < 0.001) and AIS (*r* = −0.111, *P* < 0.001). Similar inverse associations were observed for PA intensity (with improper sitting posture: *r* = −0.075, *P* < 0.001; with AIS: *r* = −0.101, *P* < 0.001), duration (−0.113 and −0.069, respectively; both *P* < 0.001), and frequency (−0.086 and −0.090, respectively; both *P* < 0.001). In contrast, improper sitting posture was positively associated with AIS (*r* = 0.127, *P* < 0.001).

**Table 5:** Association analysis of physical activity and improper sitting posture with adolescent idiopathic scoliosis.

Variables	Physical activity	Intensity of physical activity	Duration of physical activity	Frequency of physical activity	Improper sitting posture	Adolescent idiopathic scoliosis
Physical activity	1					
Intensity of physical activity	0.684***	1				
Duration of physical activity	0.804***	0.478***	1			
Frequency of physical activity	0.570***	0.250***	0.402***	1		
Improper sitting posture	−0.100***	−0.075***	−0.113***	−0.086***	1	
Adolescent idiopathic scoliosis	−0.111***	−0.101***	−0.069***	−0.090***	0.127***	1

*** *p* < 0.001, and so on.

**Figure 2:**
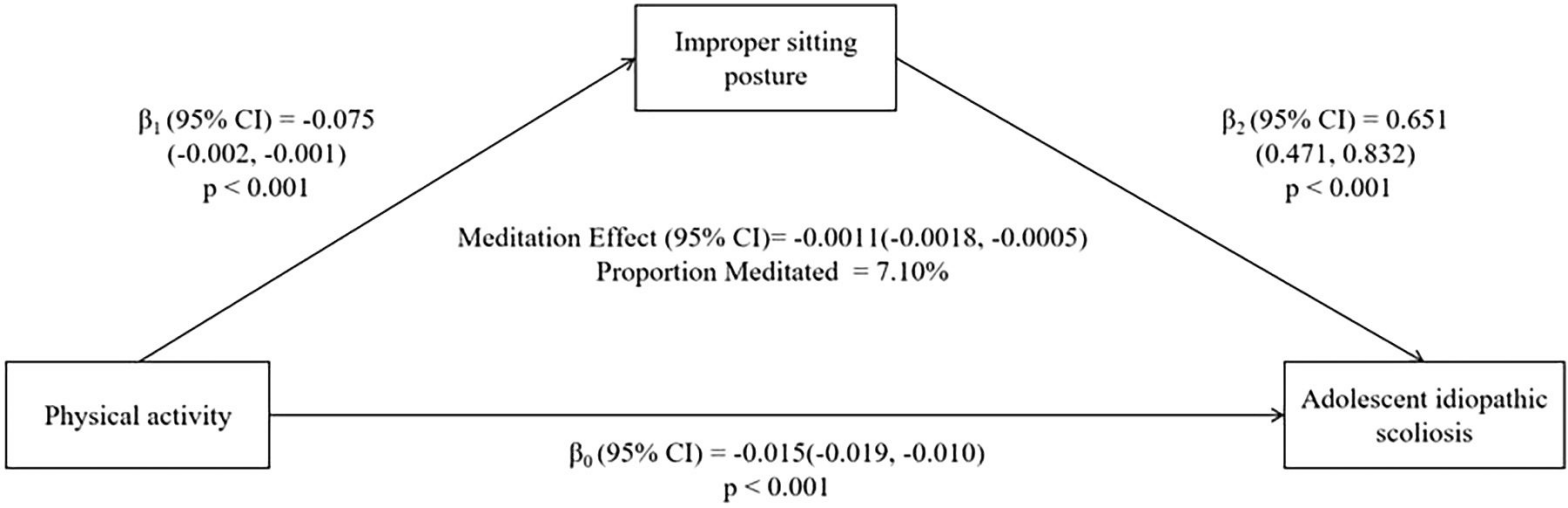
Conceptual framework for the cross-sectional mediation model. *β*0 represents the total effect of physical activity on AIS. *β*_1_ represents the effect of physical activity on adolescents’ improper sitting posture; *β*_2_ represents the effect of improper sitting posture on AIS. The mediating effect was calculated as the product of “*β*_1_” and “*β*_2_” (*β*_1_ × *β*_2_), and the mediating ratio was calculated as the ratio of the mediating effect product to the total effect [(*β*_1_ × *β*_2_)/*β*_0_]. AIS, adolescent idiopathic scoliosis.

PA exhibited a statistically significant negative total effect on AIS (*β* = −0.015, 95% CI: −0.019 to −0.010; *P* < 0.001). Improper sitting posture partially mediated this association, with an indirect effect of −0.0011 (95% CI: −0.0018 to −0.0005), accounting for 7.10% of the total effect. The indirect pathway from PA to AIS through improper sitting posture is depicted in Figure 2.

Improper sitting posture significantly mediated the associations between each PA dimension and AIS: intensity (indirect effect = −0.0138, 95% CI: −0.0246 to −0.0048, proportion mediated = 6.44%), duration (−0.0251, 95% CI: −0.0388 to −0.0144, 16.17%), and frequency (−0.0218, 95% CI: −0.0346 to −0.0109, 10.43%) in Supplementary Figure 1–S3.

## Discussion

This study, based on data of Chinese adolescents from 2021, indicates that PA was negatively associated with improper sitting posture and AIS, while improper sitting posture was positively associated with AIS. Improper sitting posture played a partial mediating effect between PA and AIS. Subgroup analyses revealed that BMI significantly influenced the association between improper sitting posture and AIS, thereby confirming our initial hypothesis.

Previous studies have demonstrated that lower PA levels linked to a higher prevalence of AIS (23). In addition, more frequent sports participation was associated with a lower risk of AIS progression (24). In the present study, we confirmed these findings and observed a negative association between PA and AIS (OR = 0.985, 95% CI: 0.981–0.990, *P* < 0.001). Higher levels of PA may reduce the risk of AIS by enhancing neuromuscular coordination and paraspinal muscle symmetry, as AIS have been shown to exhibit asymmetric trunk muscle activation and impaired spinal stabilization, which may alter vertebral loading patterns and compromise postural control during spinal growth (25). PA may also improve vestibular and sensorimotor integration involved in postural regulation, because abnormalities in balance control and vestibular processing have been identified in AIS, while neuromuscular stimulation and repetitive movement training may enhance central nervous system adaptation and improve dynamic spinal stability under mechanical loading conditions (26). Greater PA may increase the load-bearing and fatigue-resistance capacity of paraspinal musculature, thereby reducing asymmetric spinal loading and vertebral rotational stress (27). These findings support the view that PA is an important behavioral factor in adolescent spinal health, and that increasing appropriate PA may help reduce the risk of AIS.

Previous studies have indicated that higher levels of PA were linked to better postural habits and reduced prevalence of postural deviations in school-aged children (28), while another study found that increased participation in moderate-to-vigorous PA was associated with lower sedentary time and fewer unhealthy sitting behaviors (29). In the present study, we extended these findings by demonstrating a negative association between PA and improper sitting posture. Higher levels of PA may reduce the risk of improper sitting posture by enhancing neuromuscular coordination and trunk muscle endurance, as activation and co-contraction of deep stabilizing muscles such as the transvs. abdominis and multifidus are essential for and upright postural alignment during prolonged sitting (30). PA may also improve proprioceptive feedback and sensorimotor integration involved in postural regulation, because regular movement stimulation enhances afferent input from muscle spindles and joint mechanoreceptors, thereby improving central nervous system control of body alignment and reducing postural collapse under sustained sitting conditions (31). Higher levels of PA may reduce improper sitting posture by decreasing prolonged sedentary exposure, thereby limiting continuous static flexion loading and neuromuscular fatigue of trunk stabilizing muscles, which are known to impair postural control and promote slumped sitting patterns during sustained sitting tasks (32). These neuromuscular adaptations and behavioral patterns may reduce the likelihood of adopting and maintaining improper sitting posture over time.

Previous studies have indicated that adolescents with sustained slumped sitting posture exhibited greater spinal curvature deviations compared with those maintaining neutral alignment (33). In addition, prolonged asymmetric sitting is linked to increased trunk asymmetry and a higher likelihood of scoliosis-related postural abnormalities (34, 35). In the present study, we confirmed these findings and demonstrated a positive association between improper sitting posture and AIS (OR = 1.989, 95% CI: 1.664–2.376, *P* < 0.001). Furthermore, improper sitting posture partially mediated this relationship between PA and scoliosis, with a mediation effect of −0.0011 (95% CI: −0.0018 to −0.0005), representing 7.10% of the overall effect variability. Prolonged asymmetric sitting posture may contribute to the development and progression of AIS by sustaining paraspinal muscle imbalance and altering proprioceptive integration, which can impair central postural regulation and reduce the ability to maintain symmetrical spinal alignment during adolescent growth (36). Persistent maladaptive sitting posture may also accelerate AIS progression by increasing asymmetric compressive and dorsal shear forces on immature vertebral structures, thereby promoting vertebral rotation and uneven spinal growth through mechanically induced modulation of spinal development (27). Prolonged slumped or asymmetric sitting also fatigues deep trunk stabilizers and alters spinal-pelvic alignment—reducing segmental stability and increasing cumulative mechanical stress on discs, ligaments, and paraspinal tissues, thereby accelerating AIS (37). Given that adolescents spend a substantial proportion of their daily time in seated activities, these cumulative effects may progressively contribute to the initiation or progression of spinal deformities. These findings support the role of improper sitting posture as a significant behavioral risk factor for AIS and highlight its potential involvement in the pathway association PA to spinal health.

Our subgroup analysis revealed that BMI significantly modified the association between improper sitting posture and AIS, with overweight adolescents showing a stronger positive association. This modifying effect may be explained by biomechanical mechanisms: higher body mass increases gravitational loading on the spine during sitting, which may amplify the asymmetric mechanical strain already imposed by maladaptive postural alignment on immature vertebral structures, thereby rendering overweight adolescents particularly susceptible to the spinal effects of improper sitting posture (38, 39).

This study provides practical public health implications for adolescent musculoskeletal health. Our findings suggest that promoting adequate PA and improving sitting posture may represent effective strategies for reducing the risk of AIS. Public health efforts should prioritize school-based interventions that encourage regular PA and proper sitting habits during prolonged classroom activities. These results underscore the importance of integrating posture education and PA promotion into school health programs to support healthy spinal development in adolescents.

Future research should prioritize longitudinal and causal designs to disentangle the directionality between PA, sitting posture, and AIS. Incorporating objective assessments of movement and posture, alongside repeated measurements over time, would improve precision and reduce bias. Mechanistic studies integrating neuromuscular function and spinal biomechanics are also needed to better explain the observed associations. Future studies should include adolescents from different regions and school environments to assess whether the observed associations are consistent across varying behavioral and environmental conditions.

## Advantages and limitations

This study possesses several notable strengths. First, the relatively large sample size enhances the statistical power and stability of the findings. Second, we simultaneously examined PA, sitting posture, and AIS within a unified analytical framework, allowing a more comprehensive understanding of their interrelationships. Third, the inclusion of subgroup analyses further strengthens the study by identifying potential effect modifiers, offering more nuanced insights into population heterogeneity.

However, several limitations warrant consideration. First, the use of self-reported measures for PA, sitting posture, and AIS may introduce recall bias and misclassification. In particular, self-reported sitting posture may be subject to social desirability bias, as adolescents tend to overreport proper behaviors when completing questionnaires about their daily habits. Second, the cross-sectional design precludes causal inference, and the temporal relationships among PA, sitting posture, and AIS cannot be determined. Third, reverse causality cannot be ruled out. Adolescents with preexisting spinal asymmetry or early-stage AIS may reduce their PA levels or adopt improper sitting posture due to physical discomfort, impaired balance control, or functional limitations, rather than the reverse. Subclinical spinal deviations that have not yet reached diagnostic thresholds may also subtly influence movement behaviors and postural choices before formal AIS identification. This bidirectional possibility complicates the interpretation of the observed associations and underscores the need for longitudinal designs to clarify the temporal sequence between PA, sitting posture, and AIS. Fourth, missing data may have introduced selection bias and affect the validity of the findings. Fifth, the total amount of PA assessed by the PARS-3 scale in this study did not distinguish the biomechanical effects of different exercise types on the spine. As AIS involves three-dimensional structural deformities, various exercise modes may impose distinct loading patterns on the spine, with asymmetric or high-impact activities potentially exacerbating spinal asymmetry while symmetric core training may confer protective effects. Future studies incorporating exercise type and spine-specific biomechanical assessments are warranted to more accurately elucidate the complex relationship between PA and AIS. Sixth, daily sleep duration was adjusted as a covariate, but its assessment was relatively crude, categorized into only three broad groups (<7 h, 7–9 h, >9 h), without capturing other important dimensions such as sleep quality, regularity, or daytime napping patterns, which may potentially confound the observed associations. Finally, although the study population was drawn from nine provinces in China with considerable regional diversity, all participants were recruited from school settings and did not include adolescents who were out of school or unable to attend due to severe physical disabilities. This may modestly limit the generalizability of our findings to the broader adolescent population. Nevertheless, given that the vast majority of adolescents in China are enrolled in compulsory education, this sampling frame is unlikely to have substantially biased the observed associations.

## Conclusion

This study employed a cross-sectional design to examine the associations among PA, sitting posture, and AIS. Our findings showed that PA was negatively associated with both improper sitting posture and AIS, while improper sitting posture was positively associated with AIS. Notably, improper sitting posture partially mediated the association between PA and AIS, suggesting a potential behavioral pathway linking movement behaviors to spinal health. These findings highlight modifiable targets for AIS prevention, particularly promoting adequate PA and improving sitting posture in adolescents.

## Data Availability

The original contributions presented in the study are included in the article/Supplementary Material, further inquiries can be directed to the corresponding author/s.
