# Supplementary material for: The association between physical activity and adolescent idiopathic scoliosis: improper sitting posture as a mediator
Source: Front Pediatr. 2026 Jul 31;14:1884485. doi: 10.3389/fped.2026.1884485 (PMC13473334; doi:10.3389/fped.2026.1884485)
Supplement: Supplementary file 1 [file Supplementaryfile1.docx]

Supplementary Table 1 Subgroup analysis of physical activity on adolescent idiopathic scoliosis

| Variables | *N* | Adolescent idiopathic scoliosis | *P for interaction* |
| --- | --- | --- | --- |
| Sex |  |  | 0.145 |
| Male | 1790 | 68(3.8) |  |
| Female | 1746 | 95(5.4) |  |
| Age |  |  | 0.069 |
| 10–12 years old | 1074 | 38(3.5) |  |
| 13–15 years old | 2000 | 108(5.4) |  |
| 16–19 years old | 462 | 17(3.7) |  |
| BMI |  |  | 0.026 |
| Underweight | 770 | 13(1.7) |  |
| Normal weight | 1630 | 72(4.4) |  |
| Overweight | 740 | 63(8.5) |  |
| Obese | 396 | 15(3.8) |  |
| Daily sleep duration |  |  | 0.675 |
| ＜7h | 1050 | 54(5.1) |  |
| 7–9h | 2242 | 102(4.5) |  |
| ＞9h | 244 | 7(2.9) |  |
| Abbreviations: BMI, body mass index. | | | |

Supplementary Table 2 Subgroup analysis of physical activity on improper sitting posture

| Variables | *N* | Improper sitting posture | *P for interaction* |
| --- | --- | --- | --- |
| Sex |  |  | 0.021 |
| Male | 1790 | 1.96±0.51 |  |
| Female | 1746 | 1.96±0.48 |  |
| Age |  |  | 0.597 |
| 10–12 years old | 1074 | 1.84±0.51 |  |
| 13–15 years old | 2000 | 2.01±0.47 |  |
| 16–19 years old | 462 | 2.03±0.52 |  |
| BMI |  |  | 0.033 |
| Underweight | 770 | 1.93±0.54 |  |
| Normal weight | 1630 | 1.99±0.49 |  |
| Overweight | 740 | 1.94±0.46 |  |
| Obese | 396 | 1.93±0.45 |  |
| Daily sleep duration |  |  | 0.023 |
| ＜7h | 1050 | 2.03±0.51 |  |
| 7–9h | 2242 | 1.95±0.46 |  |
| ＞9h | 244 | 1.77±0.62 |  |
| Abbreviations: BMI, body mass index. | | | |

Supplementary Table 3 Subgroup analysis of improper sitting posture on adolescent idiopathic scoliosis

| Variables | *N* | Adolescent idiopathic scoliosis | *P for interaction* |
| --- | --- | --- | --- |
| Sex |  |  | 0.066 |
| Male | 1790 | 68(3.8) |  |
| Female | 1746 | 95(5.4) |  |
| Age |  |  | 0.023 |
| 10–12 years old | 1074 | 38(3.5) |  |
| 13–15 years old | 2000 | 108(5.4) |  |
| 16–19 years old | 462 | 17(3.7) |  |
| BMI |  |  | 0.008 |
| Underweight | 770 | 13(1.7) |  |
| Normal weight | 1630 | 72(4.4) |  |
| Overweight | 740 | 63(8.5) |  |
| Obese | 396 | 15(3.8) |  |
| Daily sleep duration |  |  | 0.056 |
| ＜7h | 1050 | 54(5.1) |  |
| 7–9h | 2242 | 102(4.5) |  |
| ＞9h | 244 | 7(2.9) |  |
| Abbreviations: BMI, body mass index. | | | |

Supplementary Figure 1. **Conceptual framework for the cross-sectional mediation model.** β_0_ represents the total effect of intensity of physical activity on AIS. β_1_ represents the effect of intensity of physical activity on adolescents' improper sitting posture; β_2_ represents the effect of improper sitting posture on AIS. The mediating effect was calculated as the product of "β_1_" and "β_2_" (β_1_×β_2_), and the mediating ratio was calculated as the ratio of the mediating effect product to the total effect [(β_1_×β_2_) /β_0_]. Abbreviations: AIS, adolescent idiopathic scoliosis.

Supplementary Figure 2. **Conceptual framework for the cross-sectional mediation model.** β_0_ represents the total effect of duration of physical activity on AIS. β_1_ represents the effect of duration of physical activity on adolescents' improper sitting posture; β_2_ represents the effect of improper sitting posture on AIS. The mediating effect was calculated as the product of "β_1_" and "β_2_" (β_1_×β_2_), and the mediating ratio was calculated as the ratio of the mediating effect product to the total effect [(β_1_×β_2_) /β_0_]. Abbreviations: AIS, adolescent idiopathic scoliosis.

Supplementary Figure 3. **Conceptual framework for the cross-sectional mediation model.** β_0_ represents the total effect of frequency of physical activity on AIS. β_1_ represents the effect of frequency of physical activity on adolescents' improper sitting posture; β_2_ represents the effect of improper sitting posture on AIS. The mediating effect was calculated as the product of "β_1_" and "β_2_" (β_1_×β_2_), and the mediating ratio was calculated as the ratio of the mediating effect product to the total effect [(β_1_×β_2_) /β_0_]. Abbreviations: AIS, adolescent idiopathic scoliosis.
